# The role of miRNA-210 in pre-eclampsia development

**DOI:** 10.1080/07853890.2022.2071459

**Published:** 2022-05-11

**Authors:** Ilona Jaszczuk, Dorota Koczkodaj, Adrianna Kondracka, Anna Kwaśniewska, Izabela Winkler, Agata Filip

**Affiliations:** a Department of Cancer Genetics with Cytogenetic Laboratory, Medical University of Lublin, Lublin, Poland; b Department of Obstetrics and Pathology of Pregnancy, Medical University of Lublin, Lublin, Poland; c Second Department of Gynecological Oncology, St. John’s Center of Oncology of the Lublin Region, Lublin, Poland

**Keywords:** miR-210, hypoxia, pre-eclampsia

## Abstract

MicroRNAs (miRNAs) are a class of small non-coding, single-stranded RNAs (ribonucleic acids) that play important roles in many vital processes through their impact on gene expression. One such miRNA, miR210, represents a hypoxia-induced cellular miRNA group that hold a variety of functions. This review article highlights the importance of miR-210 in the development of pre-eclampsia.

**KEY MESSAGE**
- miR-210 is a promising biomarker for monitoring pregnancy with pre-eclampsia. Overexpression of miR-210 had a negative impact on the process of cell migration and trophoblast invasion.

## 1. Introduction

MicroRNAs (miRNAs) are a class of non-coding, single-stranded RNAs consisting of 19–25 nuceotides [1]. They play important and indisputable roles in post-transcriptional regulation of gene expression *via* a process called “silencing”. The synthesis of miRNAs in the cell is highly-orchestrated. It consists of three steps which take place in the cell nucleus and in the cytoplasm. Mature miRNAs are functionally part of the RNA-induced silencing complex (RISC), which contains besides miRNAs, proteins from the Argonaute family (Ago2).

Post-transcriptional regulation of the genes expression using the RISC complex is based on the complementarity of 2–8 nucleotides of the “seed region” in the mature miRNAs and the 3′ unstranslated region (3’UTR) of the transcript [2]. Research by Broughton et al. [3] showed, however, that miRNA interactions with other gene regions including the 5 'UTR, coding sequence and promoters provide the information necessary for stable and specific miRNA-target interactions *in vivo*. It should be noted that miRNAs inhibit the translation of messenger RNA (mRNA) or facilitate the cleavage of target mRNA [4]. The current 22.1 release of miRNA database miRBase includes 38,589 mature miRNA sequences [5].

A single miRNA can possibly regulate the expression of multiple genes. Conversely, the expression of a single gene can be regulated by different miRNAs. Expression of over 60% of all human genes is regulated by miRNAs. It has been shown that microRNAs play a key role in the course of many physiological processes, including organism growth, cell maturation and specialisation, cell migration and apoptosis, and even the development of pregnancy. In addition, microRNAs also participate in the development of many pathological processes: neoplastic transformation, inflammatory processes and the development of chronic diseases.

Due to their occurrence and the possibility of obtaining them for research and analysis, miRNAs have been divided into tissue-specific and circulating. The latter can be measured in physiological fluids (including blood serum), excreta and secretions [6]. It has also been shown that miRNA particles circulating in the serum are very stable [7].

In our study, we would like to focus on miR-210 and its role in the development of pre-eclampsia. A circulating, placental-derived microRNA, miR-210, due to the induction of hypoxia disorders, is a root cause of the damage to the vascular endothelium in the trophoblast during the development of pre-eclampsia. The role of miR-210 seems to be of greatest importance in the regulation of cell division, in the response to DNA damage, in mitochondrial oxidative metabolism and in angiogenesis [8]. The biogenesis of miR-210 and its importance at the cellular level make it a good candidate for prognostic purposes and therapeutic intervention in the future.

## 2. Pre-eclampsia

According to published recommendations of the World Health Organisation (WHO), pre-eclampsia is a severe multifactorial complication in obstetrics, affecting 2–8% of all pregnancies. Pre-eclampsia is defined as co-occurrence of hypertension (blood pressure >140/90 mmHg) and proteinuria (>0.3 g of protein in the 24-h collection of urine) after 20 weeks of gestation, in a previously normotensive woman [9].

Clinically, pre-eclampsia affects both the growing foetus and the pregnant woman [10]. The sequelae of developing pre-eclampsia can appear in the foetus earlier than in the second trimester, leading to intrauterine growth restriction (IUGR), intrauterine hypoxia, preterm labour [11] or cardiovascular complications in the offspring in the perinatal period and beyond [12]. The most serious complication of pre-eclampsia is intrauterine death of the foetus and increased perinatal mortality of the newborn and the mother [13]. The maternal form of PE is observed most often after 34 weeks of pregnancy. Significant risk factors for the development of PE are metabolic disorders: insulin resistance, obesity, diabetes, chronic hypertension, dyslipidemia, hyperhomocysteinemia, autoimmune diseases and thrombophilia [14].

There are many theories that attempt to explain the pathogenesis of PE. Abnormal immune response in pregnant women to paternal haplotype of the foetus (immune factor), abnormal trophoblast invasion, factors causing vascular endothelial damage and insufficient placental perfusion leading to hypoxia, imbalance between the level of prostacyclin and tromboxane and maternal systemic inflammatory response are the most frequently mentioned factors contributing to PE development. The common denominator for most theories is the vascular factor that is associated with endothelial damage or vascular spasm [15,16].

Due to the multifactorial conditioning of pre-eclampsia, attempts were made to determine the risk of developing PE in women with a positive family history, estimating it at 2,9% (95%, CI 1.7–4.9) [17].

Pre-eclampsia is recognised after 20 weeks of gestation, but the first unfavourable factor leading to its development is incorrect implantation [15]. The solid shell, formed by the trophoblast cells at the end of the 3rd week after fertilisation, protects the embryo against the adverse effects of increased oxygen levels and xenobiotics, which could disturb organogenesis [18]. On the other hand, growth factors contained in the secretions of the endometrial glands stimulate the development of the trophoblast [19] and a kind of dialogue arises between the decidua and the endometrial glands [20,21]. Disturbance of the balance between cytotrophoblast cells and the activity of endometrial glands may lead to improper embryo implantation, and, as a consequence, to many complications of pregnancy, including the development of pre-eclampsia.

The solid shell of the developing trophoblast is also the source of the EVT (extra-villi trophoblast cells) necessary for the remodelling of the maternal spiral arteries. In normal pregnancies, EVT is responsible for destroying the smooth muscle and elastin in the walls of the spiral arteries and replacing them with inert fibrinoid material [22]. The described remodelling is crucial for changes in the placental circulation, which reduces the velocity and pulsatility of the influencing maternal blood and protects the delicate placental villi and microvilli from damage.

In the histopathological examination of placenta from complicated pregnancies with pre-eclampsia, abnormal remodelling of maternal spiral arteries as a result of implantation disorders and other arterial changes, acute atherosclerosis with fibrinoid necrosis and accumulation of lipid-laden intimal macrophages were noticed [23]. Co-occurring coagulation disorders may additionally cause decrease uteroplacental blood flow and subsequent foetal hypoxia.

In addition, an overgrowth of syncytiotrophoblasts with degeneration or apoptosis of some of them has been observed. This leads to the release of trophoblastic debris [24], cell-free DNA [25], exosome, pro-inflammatory factors [26] and anti-angiogenic factors into the systemic circulation of pregnant women. At the molecular level, during oxidative stress, the activation of hypoxia-dependent gene expression regulation mechanisms in trophoblast cells was observed. Both exosomes contain different miRNAs and some of the placenta-derived circulating miRNAs are considered important and accessible biomarkers of pre-eclampsia development [27].

**Figure 1.**
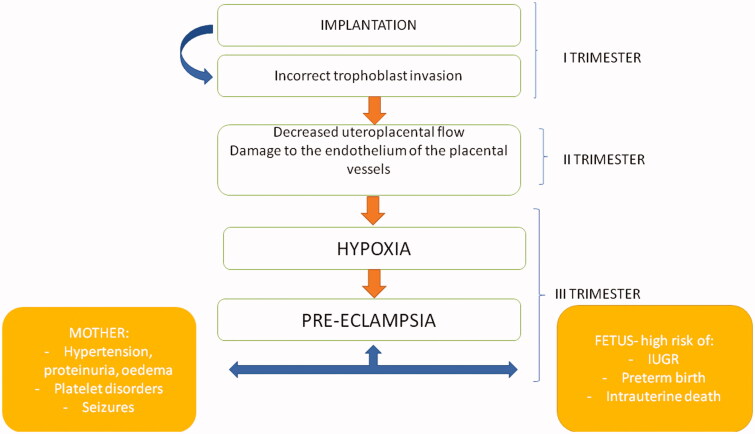
The impact of incorrect trophoblast implantation and vascular complications on pre-eclampsia development.

Placental stress leads also to a systemic inflammatory response and dysfunctions of periferal endothelial cells in pregnant women who develop pre-eclampsia. Among the potential molecular mediators of pre-eclampsia, the sFLT (soluble receptor for vascular endothelial growth factor VEGF) and PlGF (placental growth factor) deserve special attention. The increasing level of sFLT binds VEGF more strongly, reducing its bioavailability for the maternal endothelium, and the decrease in endoegenic nitric oxide production leads to vasoconstriction [28] (Figure 1).

## 3. miR-210

miR-210 represents a hypoxia-induced miRNA group, also known as hypoxamiRs, found in normal and transformed cells in response to the hypoxic state [29, 30–33]. Studies on miR-210 biogenesis indicate its involvement in arresting cell proliferation, inhibiting mitochondrial respiration, arresting DNA repair and angiogenesis. Changes in miR-210 expression have been demonstrated in many diseases such as tumour progression, myocardial infarction, and ischaemic skin wounds. Pineles et al. analysed 157 miRNAs from PE and SGA patients and found that miR-210 levels were higher in placentas and sera than controls, making miR-210 a promising biomarker for pre-eclampsia development [34]. It is expressed in both villous and extravillous trophoblast and could be measured in the serum of pregnant women [35].

Hypoxia is an oxygen deficiency condition that significantly affects cell metabolism, and hypoxia-induced factors (HIFs) are the most sensitive cellular sensors of hypoxia. HIFs are heterodimers, consisting of HIFα (oxygen-sensitive α-subunit: HIF-1α, HIF-2α, HIF-3α) and HIF1β (constitutively expressed β-subunit) [29]. During hypoxia HIFα is stabilised by heterodimerization with the β-subunit. Heterodimers of HIFs enter the cell nucleus and activate genes involved in cell proliferation, differentiation and apoptosis, angiogenesis or erythropoesis. Most noted genes that are activated during hypoxia with HIFs regulation include glucose transporter 1 (*GLUT1*), vascular endothelial growth factor (*VEGF*) and erythropoetin (*EPO*) [35].

In studies on the influence of hypoxia on cellular metabolism and changes in the expression of genes, the importance of miRNAs is emphasised. In most analyses, researchers have noted an increase of miR-210 level in hypoxic tissue in the HIF-dependent or HIF-independent ways [35].

The relationship between oxygen depletion, stabilisation of HIFs heterodimers and enhancement of nuclear gene expression lies at the basis of transcription activation of miR-210 genes. Subunit HIF-1α recognises and directly binds to a selected HRE region (hypoxia responsive element) on the proximal miR-210 promoter. The sequence of HRE is highly conserved in various species and proves the importance of the synthesis of miR-210 in the hypoxic state [31].

## 4. Discussion

**Figure 2.**
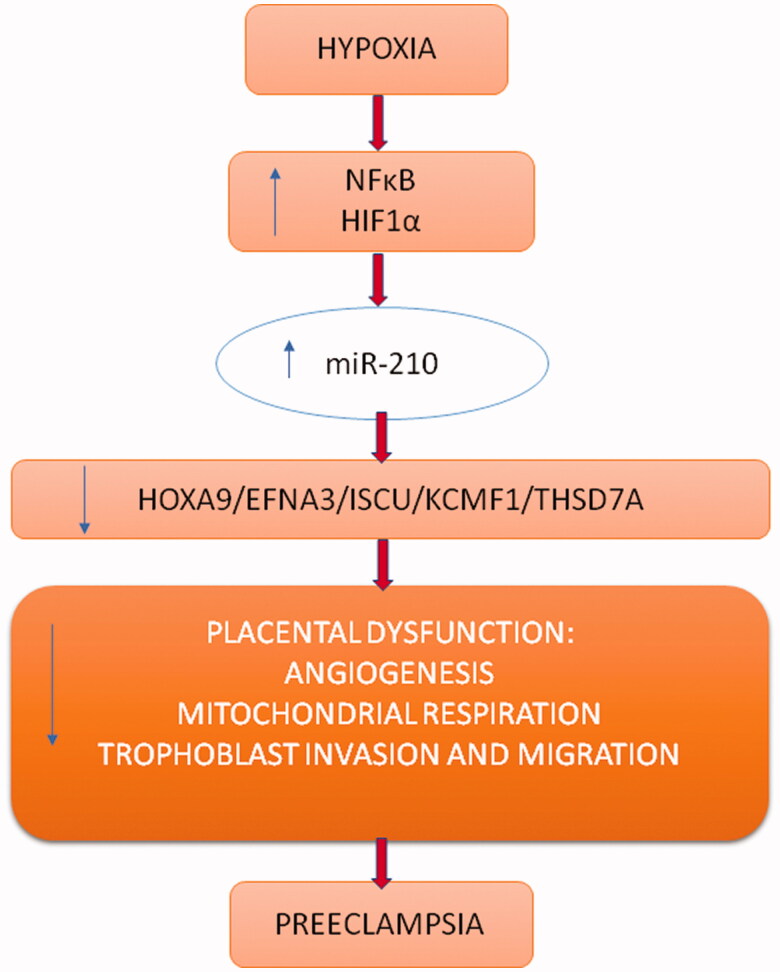
Regulation of expression of selected genes by miR-210 under hypoxic condition.

The relationship between the development of PE and changes in the expression level of tissue-specific and circulating miRNAs is indisputable. (Table 1) One of the more commonly miRNAs correlated with PE is miR-210, a well-known a hypoxia-response miRNA. The importance of miR-210 in the pathogenesis of PE is still the subject of much research that allow the identification of a wide variety of miR-210 target genes (Figure 2).

**Table 1.** Importance of selected miRNAs in the development of pre-eclampsia [36,37].

First stage	Impaired trophoblast migration and invasion	miR-195	Downregulated	[38]
		miR-210	Upregulated	[34,39]
		miR-155	Upregulated	[40]
	Impaired angiogenesis	miR-210	Upregulated	[41]
		miR-16	Upregulated	[42]
		miR-26b	Upregulated	
		miR-29b	Upregulated	
		miR-181a	Upregulated	
		miR-195	Upregulated	
		miR-222	Upregulated	
		miR-335	Upregulated	
		miR-15b	Upregulated	[43]
		miR-126	Downregulated	[44]
Second stage	Dysregulation of maternal immune system	miR-223	Downregulated in placenta	[38]
			Upregulated in plasma	[45]
		miR-210	Upregulated	[46,47]

Wang et al. indicate the existence of a CPEB2-mediated relationship between miR-210 and HIF-1α in the human trophoblast, especially within the villous trophoblast. Therein, *CPEB2* (cytoplasmic polyadenylation element-binding 2) is a direct target of miR-210. Under the influence of hypoxia, the HIF-1α enhances the expression of miR-210. CPEB2 can inhibit the HIF-1α translation by binding a cytoplasmic polyadenylation element (CPE) to the 3’UTR of HIF-1α mRNA. However, in this study, the levels of CPEB2 in the pre-eclamptic placenta were evidently lower and the levels of miR-210 expression higher. CPEB2 is a significant factor in trophoblast syncytialization, which is blocked by increasing levels of miR-210, as observed in the trophoblast during pre-eclampsia development [39].

Overexpression of miR-210 as the response to hypoxia, alters extravillous trophoblast (EVTs) in the first trimester by modulating mitochondrial function. Mitochondrial dysfunction may increase the level of reactive oxygen species (ROS), leading to increased oxidative stress. Upregulation of ROS stabilises HIFs, hence, indirectly stimulating the expression of miR-210, which inhibits EVTs invasion in the first trimester. Target genes for miR-210 listed by Anton et al. are *NDFUA4, SDHD* and *ISCU,* and the effect upon them is a decrease in mitochondrial respiration, a decrease in ATP synthesis and an increase in ROS, which can also directly damage trophoblast cells [48].

Luo et al., using bioinformatics analysis, selected the thrombospondin type I domain containing (*THSD7A*) as another target gene for miR-210. Expression of TSHD7A was confirmed in placenta vasculature and in human umbilicavein endothelial cells (HUVECs). They also showed that hypoxia influences the increase in expression of miR-210 and simultaneously, induces THSD7A repression. This phenomenon was seen in the trophoblast cells of women with pre-eclampsia [49]. In addition, higher levels of miR-210 can lead to down-regulation of other genes participating in migration and trophoblast invasion, among others: *EFNA3, HOXA9* and *KCMF1* [33,50,51].

Other studies highlight the relationship between miR-210 and VGFR signalling in different cell types [52]. Accordingly, VEGF dramatically increases miR-210 expression, and miR-210, subsequently, promotes the pro-angiogenic effects of VEGF. Evidence for this claim lies in the observation that an increase in the level of sFLT, a soluble receptor for VEGF and PlGF, was observed in the pre-eclampsic placenta. It should be noted that reduced bioavailability of VEGF leads to vasoconstriction within the placenta, contributing to hypoxia. Moreover, in pregnancies complicated by pre-eclampsia, decreased levels of PlGF has been confirmed [52].

In searching for factors sensitive to hypoxia that are independent of HIFs, it was noticed that NFκB (nuclear factor κB) and Akt activation influence the accumulation of miR-210 during oxygen deficiency [53]. In turn, miR-210 can significantly affect proliferation, migration and trophoblast invasion by supression of *PTPN2* expression, which is its other target gene, in the PDGFR-Akt pathway [54].

Zhang et al. and Anton et al. emphasise the negative impact of miR-210 overexpression on the process of cell migration and trophoblast invasion [29,33]. Anton et al. also demonstrated that miR-210 inhibits trophoblast invasion *via* the MAPK signalling pathway. This is indicated by the activation of the ERK/MAPK signalling pathway and the stimulation of miR-210 expression by hypoxia and lipopolysaccharide (LPS) [29]. Beyond the aforementioned, the expression of miR-210 can contribute to PE development by interfering with potassium channel modulatory factor 1- mediated signalling in the placenta [55].

The expression level of miR-210 measured in the serum of pregnant women is clearly higher in the severe than in the mild form of PE [56]. For early-onset severe PE with IUGR, attention was drawn to poor maternal-foetal compatibility (“immune PE”) [57–59]. *CSF1* and *ITGAM* genes are also targeted by miR-210. These are mainly expressed in immune cells: phagocytes, or monocytes, granulocytes and macrophages [46,47].

In an attempt to find further correlations between the miR-210 expression level and the severity of the clinical course of pre-eclampsia. Jairajpuri et al. [47] selected 2 genes *ACVR1B* and *ADAM-17*, the down-regulation of which occurred in severe PE and was not recorded in mild PE. *ACVR1B* (activin A receptor type 1B) encodes the receptor complex with activin involved, inter alia, in the production of extracellular matrix and immunosuppression [46,47]. *ADAM-17* is a metalloproteinase domain gene involved in regulating the activity of placental cells.

Due to the multi-organ damage observed in women with pre-eclampsia, the possibility of analysing prognostic factors not only in serum, but also in other body fluids has been taken into account. Indeed, Luo et al. [49] have demonstrated the presence of miR-210 in the urine of women with PE and kidney damage.

## 5. Conclusions

Circulating miRNAs have shown to be promising biomarkers of various human physiological and disease states. An important feature of miRNAs is their stability in body fluids, especially serum, which makes it possible to obtain samples for further analysis in a minimally invasive manner. Accordingly, miR-210 detected in the serum of pregnant women comes mainly from the placenta, although it cannot be ignored that the endothelium, especially the damaged one, may also be the source of miR-210 in the serum. Some of the cited studies indicate its increase in the serum of pregnant women with clinical symptoms of pre-eclampsia even 8–12 weeks earlier [29]. All this makes miR-210 a promising biomarker for monitoring pregnancy in terms of the development of pre-eclampsia [60,61].

## Author contributions

I. J., D. K – conception and design; I. J., A. F., D. K., A. K., I. W. – analysis and interpretation of the data resources; I. J., A. F., D. K., A. K, I. W. – preparing of figures; I. J., A.F., I. W. – the drafting of the paper, revising it critically for intellectual content and the final approval of the version to be published supervision. All authors agree to be accountable for all aspects of the work.

## Data Availability

The processed data are available from the corresponding author I.W. upon reasonable request.

## Abbreviations

miRNA: miR-microRNA
RISC: RNA-induced silencing complex
3’UTR: 3’ unstranslated region
PE: pre-eclamsia
IUGR: intrauterine growth restriction
EVT: extravillus throphoblast cells
sFLT: soluble receptor for vascular endothelial growth factor
VEGF: vascular endothelial growth factor
PlGF: placental growth factor
HIFs: hypoxia-induced factors
HRE: hypoxia responsive element
ROS: reactive oxygen species
